# Radical Cure of Neuralgia of the Inferior Dental Nerve

**Published:** 1877-06

**Authors:** 


### ARTICLE VII.

### *Radical Cure of Neuralgia of the Lower Dental Nerve.*

On July 25, 1876, a woman was admitted into M. Terrillon's ward at the Hospital St. Antoine. Since January, 1875, she had been subject to attacks of neuralgia in the course of the right inferior dental nerve. The least touch, the action of speaking, of carrying a glass to the mouth, of mastication, increased the paroxysms of pain, which radiated along the lower jaw as far back as the ear. The patient, worn out by pain, and eating only with great apprehension, got thinner day by day, and sought assistance from surgery, as she had tried in vain all the medical resources used in similar cases.

The diagnosis was easily established. M. Terrollon was convinced that the neuralgia was simple, *i. e.*, of peripheric origin. There was no cerebral mischief, and pressure on the nerve diminished the pain. The neuralgia was localized to the inferior dental nerve along its course, for the mental nerve was the point of departure of the pain, and the contact of the teeth with cold water and food caused acute suffering. M. Terrillon resolved to divide the dental nerve after the method of M. Michel, of Nancy, an operation which has for its object the cutting of the nerve before it joins the dental trunk of the inferior maxillary. The patient now wishing to be rendered insensible, was placed in a chair before a window. M. Terrillon introduced into the mouth a double gag, which, depressing the tongue, gave room and light. A verticle incision, running from the last superior molar to the inferior, divided the buccal mucous membrane as far as the anterior border of the tendon of the temporal. With the finger and a canulated sound a way is made between the internal pterygoid and the branch of the inferior maxillary. After a little time the ridge of Spix, situated at the opening of the dental canal, is felt—the nerve is there, and M. Terrillon proceeds to divide it.

It is impossible, in spite of the employment of sponges and cold water, to see the bottom of the wound. He is prevented from taking up the nerve with a hook, and immediately performing resection, as some authors advise. "Having, then, my finger on the ridge," says M. Terrillon, " I try with a tenotome to cut the nerve trunk on the inner side of the jaw but my finger is so fast that I abandon this project, after many trials. The opening of the jaws producing tension of the internal pterygoid muscle, I remove the gag. Then, introducing the finger, I feel that it penetrates the wound more easily. I slide in the tenotome and perform section exactly on the branch rising above the ridge, without going too much in front or behind through fear of embracing the lingual in the section. At the moment of this procedure the patient uttered a slight cry, and a little jet of blood sprung into the mouth." The nerve being cut the patient felt relieved. The day after the operation it was clear that sensibility to touch, temperature, and to pain was abolished in the whole extent of the region of the chin. The patient left the hospital on August 6th, and was seen by M. Terrillon on November 18th. She had then all the appearance of health, contrasting singularly with her anæmic condition or her entrance to hospital. She declared she had not suffered any pain since the operation.

M. Terrillon offers the following observations and conclusions: (*a*) Section of the inferior dental nerve is indicated in all cases, when the neuralgia lasts many months, when it resists general medical treatment, and when the health of the patient suffers. (*b*) The operation should be performed as high as possible on the course of the nerve; thus recurrence will be less likely. (*c*) The method of Michel, consisting of section of the nerve before its entry into the dental canal by an incision made at the bottom of the mouth, should be preferred. It offers the advantages of not injuring any important part, of freedom against accident, of not leaving any external mark, and of not having been, up to the present, followed by recurrence. (*d*) This operation presents one inconvenience; it is a delicate one with all subjects, and difficult with many; but yet he can always manage to perform section. (*e*) Simple section ought to suffice in the great majority of cases. (*f*) Resection, which does not seem indispensable, still ought to be employed, with the aid of a special hook, when the wound is sufficiently large and not too dry so that the nerve can be easily seized.

Will the neuralgia recur? M. Letievant, an authority in such matters, says in his work on *Nervous Section*, 1873.: "A comparison of facts teaches us that recurrence after section of the dental nerve takes place, especially in the first days of the operation; sometimes in the first month, rarely after a longer time." Cures which have passed over the first weeks ought to be considered as definite. In the case of M. Terrillon, the recovery having now lasted more than four months, ought to be considered as definite.—*Med. Examiner*, from *Bulletin de Therapeutique.*

---

